# Massive Hematochezia from Ascending Colonic Varices

**DOI:** 10.5811/westjem.2015.4.26215

**Published:** 2015-06-22

**Authors:** Kaci E. Christian, Michael T. McCurdy, Darryn R. Potosky

**Affiliations:** * University of Maryland School of Medicine, Department of Medicine, Baltimore, Maryland; † University of Maryland School of Medicine, Division of Pulmonary and Critical Care, Baltimore, Maryland; ‡ University of Maryland School of Medicine, Division of Gastroenterology and Hepatology, Baltimore, Maryland

A 54-year-old man with a history of alcohol use presented with hematochezia and syncope. Upon arrival to the hospital, his bleeding had stopped. He was hemodynamically stable with hemoglobin of 11g/dL, international normalized ratio of 1.8 and platelets of 37K/mcL. Nasogastric aspirate found bilious gastric contents without blood. Esophagogastroduodenoscopy revealed mild gastritis without evidence of bleeding. Colonoscopy discovered a purple-colored semi-circumferential ascending colon lesion that inflated and deflated spontaneously, without arterial pulsation. The lesion would disappear completely upon deflation, except for a visible fibrin plug, indicative of recent bleeding.

Computed tomography further delineated the structure as varices, surrounding the right colon (Figure). Nearly 36 hours after presentation, the patient developed recurrent substantial hematochezia. He required intubation and massive transfusion for a hemoglobin nadir of 5g/dL. During emergent transjugular intrahepatic portosystemic shunt (TIPS) placement, large ascending colonic varices feeding from the superior mesenteric vein were confirmed and embolized. Bleeding ceased and he was ultimately discharged to alcohol rehabilitation.

Esophageal and gastric varices are frequent complications of advanced liver disease. Ectopic varices (ECV), those not found in the esophagus and stomach, are less common; reported locations of ECV include the small bowel, gallbladder, colon, and rectum.^1^ Non-cirrhotic causes of ECV include congenital venous anomalies, splenic vein thrombosis, superior mesenteric vein obstruction and congestive heart failure.^2^

Given their often-obscure location, ECV can be difficult to identify. Once located, the ideal therapeutic intervention is unknown. Endoscopic options include band ligation and injection of tissue adhesives.^1^ TIPS with angiographic variceal embolization represents another approach for portal decompression in the setting of hemorrhage.

Although scarcely reported in the literature, ECV should be considered a source of gastrointestinal bleeding, especially in a patient with liver disease. As seen in this case, colonic varices can cause profound hemorrhage, requiring prompt evaluation and intervention.

## Figures and Tables

**Figure:**
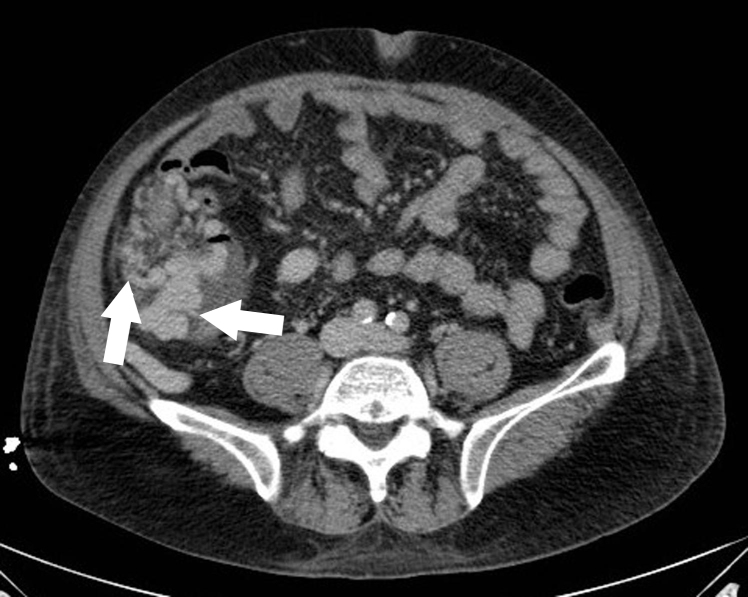
Computed tomography, axial view. Arrows indicate varices.
